# Two Cases of Pulmonary Hyalinizing Granuloma Diagnosed via Thoracoscopic Lung Biopsy

**DOI:** 10.7759/cureus.92407

**Published:** 2025-09-15

**Authors:** Madoka Goto, Yasuhisa Ichikawa, Hideki Tsubouchi, Koichi Fukumoto, Shoichi Mori

**Affiliations:** 1 Thoracic Surgery, Japanese Red Cross Aichi Medical Center Nagoya Daiichi Hospital, Nagoya, JPN

**Keywords:** hyalinizing granuloma, pulmonary hyalinizing granuloma, pulmonary resection, video-assisted thoracic surgery, wedge resection

## Abstract

Pulmonary hyalinizing granuloma is a rare, benign tumor that arises from the pulmonary parenchyma and is difficult to diagnose preoperatively and intraoperatively. These typically present on chest computed tomography images as gradually enlarging, bilateral multiple pulmonary nodules. Herein, we report two cases wherein intraoperative frozen section analysis suggested fibrosis, but histopathological examination of the surgical specimens confirmed the diagnosis of pulmonary hyalinizing granuloma. Both patients were females in their 60s with incidental chest computed tomography findings of bilateral multiple pulmonary nodules. Video-assisted thoracoscopic lung biopsy was performed, with the intraoperative frozen section analysis suggesting fibrosis. Pathological examination revealed a dense proliferation of hyalinized collagen fiber bundles, with inflammatory cell infiltration observed around the nodules, confirming a diagnosis of pulmonary hyalinizing granuloma. No nodule enlargement or emergence of new nodules was observed postoperatively after 12 months in Case 1 and 11 months in Case 2. The diagnosis of pulmonary hyalinizing granuloma is challenging both preoperatively and intraoperatively. Obtaining permanent histological specimens through lung biopsy is essential to make a definitive diagnosis.

## Introduction

Pulmonary hyalinizing granuloma (PHG) is a rare primary pulmonary nodule with an asymptomatic presentation in most cases [1]. It was first described by Engleman et al. in 1977 and has since remained an uncommon clinical entity [2]. Histologically, PHG is characterized by dense, lamellar hyaline collagen deposition surrounded by lymphoplasmacytic infiltration [2]. Radiologically, multiple nodules are observed in more than 70% of cases, and fluorodeoxyglucose uptake is frequently seen on PET-CT [1]. However, diagnosing PHG is often difficult, and pulmonary resection is usually required for a definitive diagnosis [3]. Herein, we report two cases of PHG to illustrate its clinical and diagnostic challenges.

## Case presentation

Case 1

**Figure 1:**
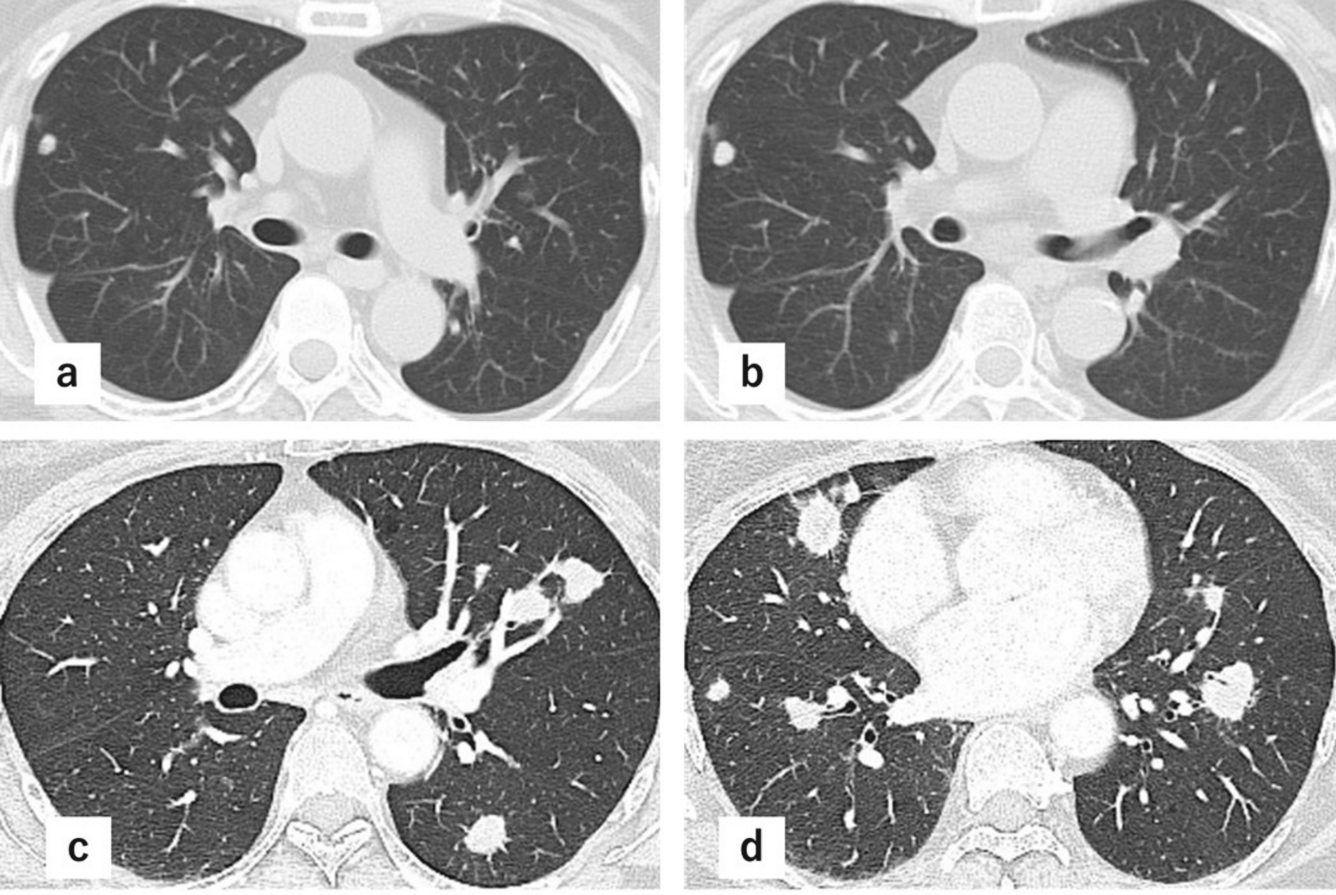
Preoperative computed tomography imaging. The computed tomography imaging findings are described as follows: Case 1 demonstrated gradually enlarging nodules from September 2020 (a) to September 2023 (b), and Case 2 demonstrated multiple bilateral pulmonary nodules measuring approximately 1-3 cm in diameter in February 2024 (c and d).

A 67-year-old female suspected of having lung cancer or a metastatic lung tumor, with a concomitant infectious disease, such as non-tuberculous mycobacterial infection or fungal infection, was referred for surgical biopsy. She had a history of pyriform sinus cancer, breast cancer, and diabetes mellitus. She had no history of respiratory infection, including tuberculosis. She underwent chemoradiotherapy for pyriform sinus cancer in 2011 and a right total mastectomy for breast cancer in 2022. During follow-up for pyriform sinus cancer in August 2013, a 3 mm right lower lobe nodule was incidentally found on CT imaging. CT follow-up was conducted every six months, and no new nodules were observed until January 2023, after which the nodules gradually increased in size and number. In September 2023, CT revealed multiple bilateral nodules measuring 0.5-1.0 cm (Figure 1, panels a-d).

**Figure 2:**
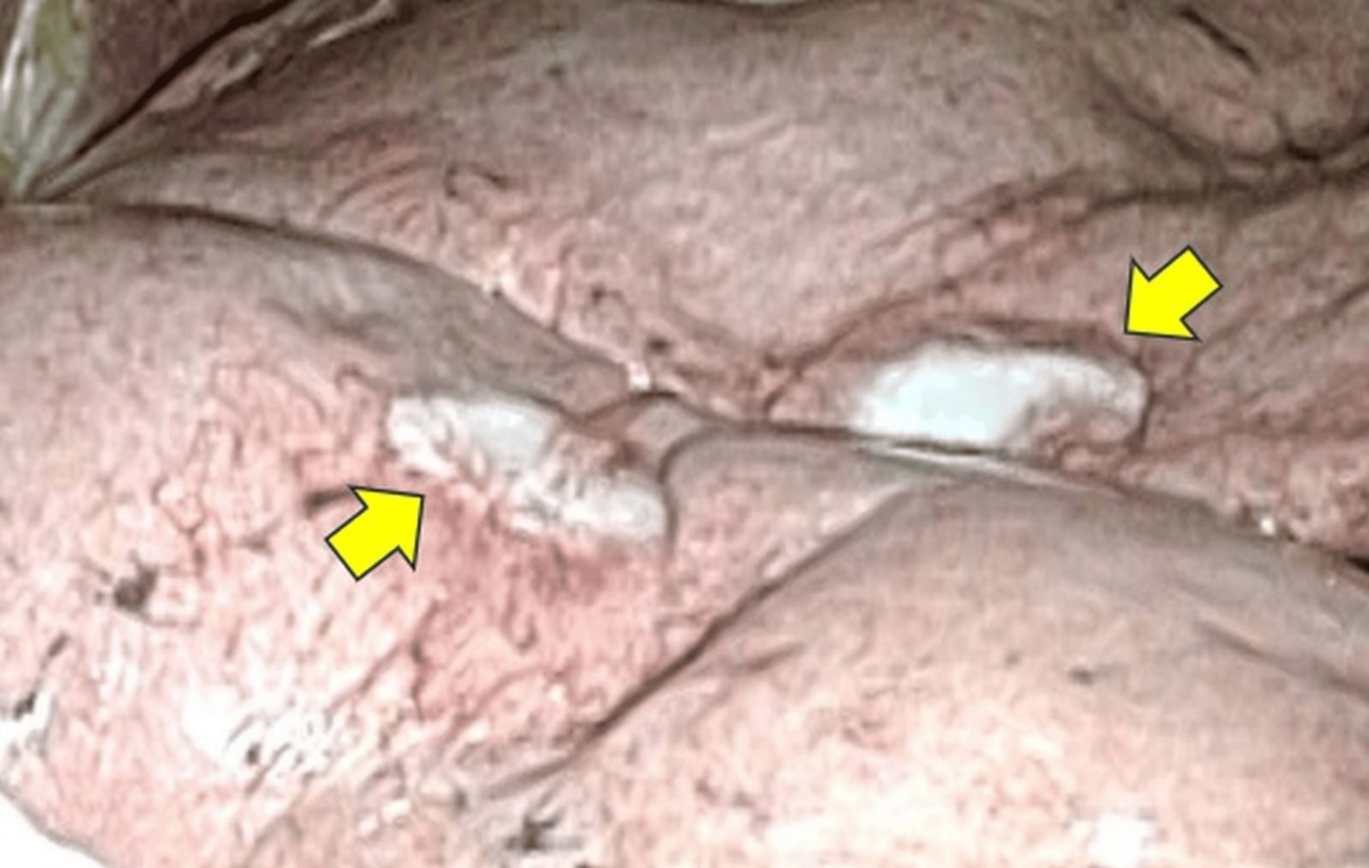
Intraoperative findings of the nodules. In Case 1, the multiple lung nodules had a grossly whitish appearance and were covered by the visceral pleura. The yellow arrows indicate pulmonary nodules.

On blood examination, tumor markers, such as carcinoembryonic antigen, cytokeratin fragment, and progastrin-releasing peptide, were not elevated. There were slight elevations in serum IgG (2,733 mg/dL) and soluble interleukin-2 receptor (928 U/mL). Antinuclear and anti-Sm antibodies were negative. The nodules and hilar lymph nodes showed no fluorodeoxyglucose (FDG) accumulation on PET-CT. To arrive at a definitive diagnosis, video-assisted thoracoscopic wedge resection of the right upper and middle lobes was performed in January 2024, revealing white, well-defined nodules (Figure 2).

**Figure 3:**
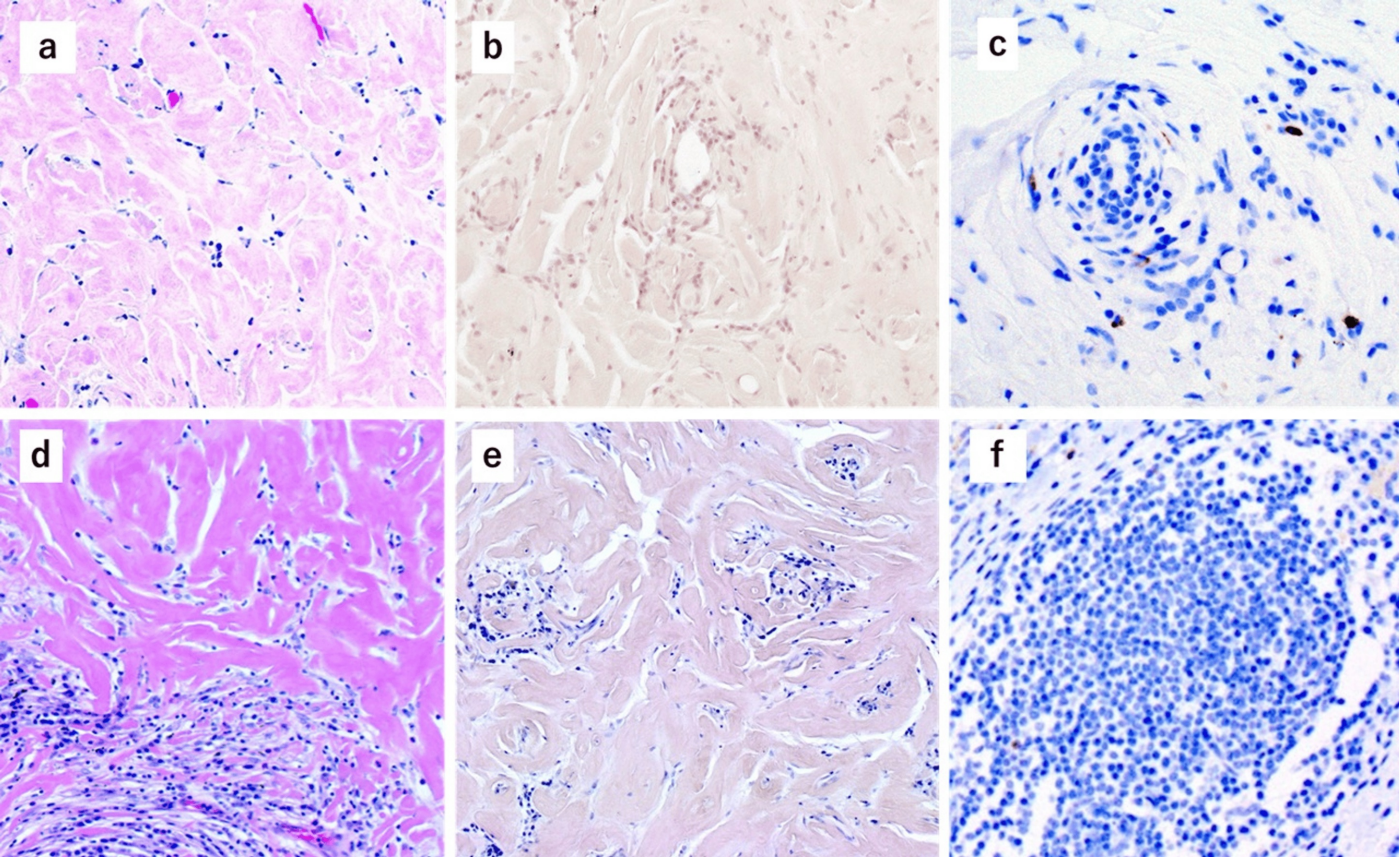
Pathological examination of the surgical specimen. Pathological examination revealed dense hyperplasia of hyalinizing collagen bundles in both Case 1 (a, ×200) and Case 2 (d, ×200). Both cases were negative on Congo red staining (b, ×200, Case 1; e, ×200, Case 2) and immunostaining for IgG4 (c, ×200, Case 1; f, ×400, Case 2).

Intraoperative frozen section revealed pulmonary fibrosis. Definitive pathological examination showed dense hyperplasia of the hyalinizing collagen bundles, which surrounded the small vessels (Figure 3, panel a). The margins of the nodule demonstrated infiltration of inflammatory cells, mainly small lymphocytes and plasma cells. To evaluate for amyloidosis and IgG4-related disease, Congo red staining and IgG4 immunostaining were performed, both yielding negative results (Figure 3, panels b and c). Finally, the patient was diagnosed with pulmonary hyalinizing granuloma (PHG). The patient was discharged on the fifth postoperative day without any complications. The patient remains alive without enlargement of the residual nodules at 12 months postoperatively.

Case 2

**Figure 4:**
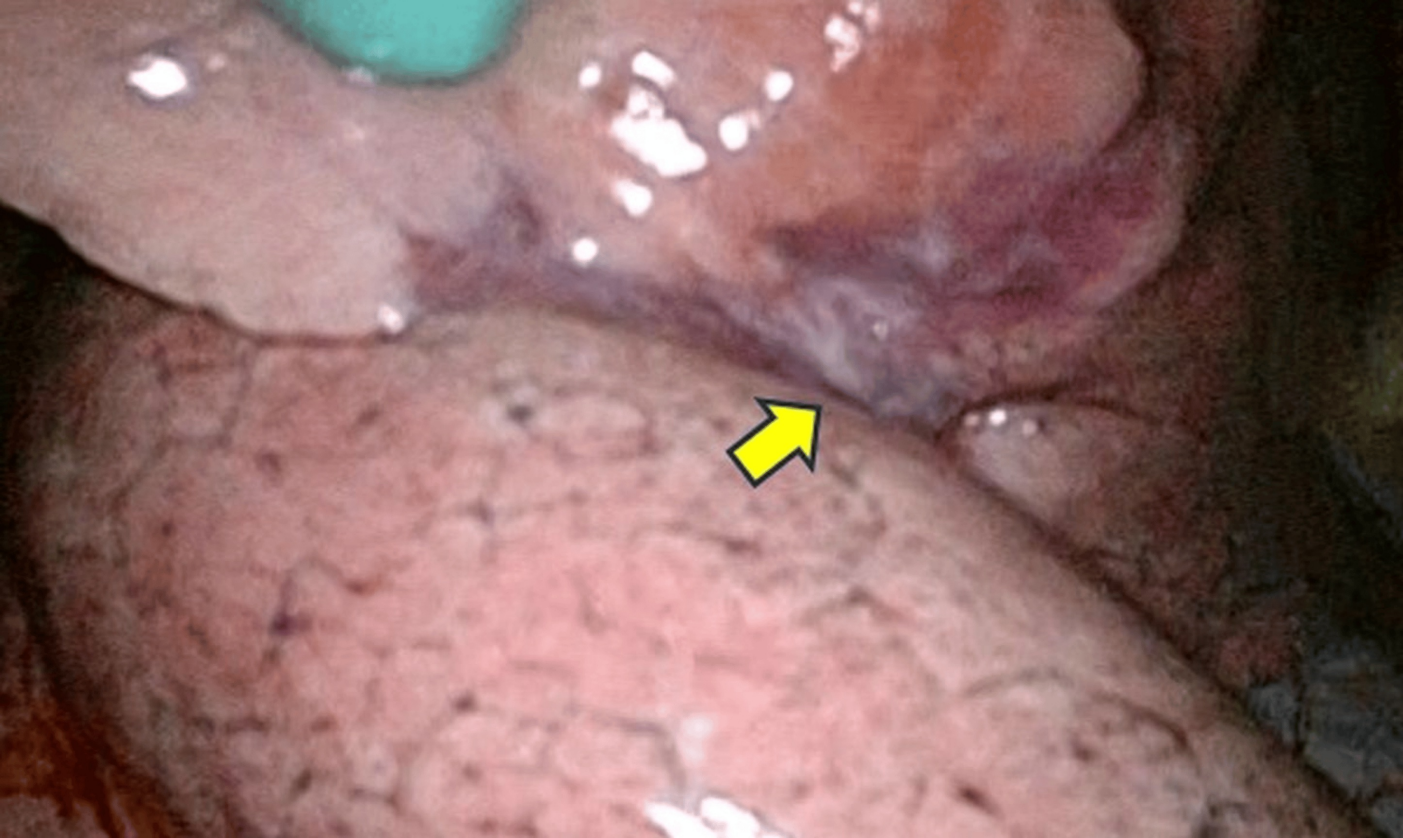
Intraoperative findings of the nodules. In Case 2, a nodule with visceral pleural indentation was observed in the right middle lobe (arrow).

A 61-year-old female with no significant medical history had an incidental finding of bilateral multiple pulmonary nodules during a routine health checkup, prompting referral to our department for diagnostic evaluation. Chest CT revealed multiple nodules measuring 1-3 cm in the bilateral lungs. FDG PET-CT demonstrated mild accumulation with a maximum standardized uptake value (SUVmax) of approximately 3. Thoracoscopic wedge resection of the right lung was performed, and intraoperative frozen section analysis suggested fibrosis (Figure 4).

Permanent pathological examination of the surgical specimen revealed dense hyperplasia of the hyalinizing collagen bundles (Figure 3, panel d). Congo red staining for amyloidosis and immunostaining for IgG4-related disease were both negative (Figure 3, panels e, f). The patient was diagnosed with PHG and remains alive at 11 months postoperatively without enlargement of the residual nodules.

## Discussion

PHG is a benign condition with a typically asymptomatic course characterized by multiple pulmonary nodules that exhibit gradual enlargement [1]. Histopathological findings include the proliferation of hyalinized collagen fiber bundles surrounded by infiltration of lymphocytes and plasma cells. PHG is differentiated from amyloid diseases through a negative Congo red staining. Although no established treatment for PHG exists, corticosteroid therapy has been successful in some reports, and its prognosis is generally favorable. Autoimmune mechanisms are suspected to be involved in PHG, although its exact etiology remains unclear. Additionally, previous reports have found that 10% of patients with PHG have concomitant infections or autoimmune diseases; however, these were not observed in the presented cases [1].

In Case 1, 10 years of prior CT follow-up data were available, enabling the radiological course to be retrospectively traced before the disease onset. Additionally, multiple pulmonary nodules accompanied by whitish pleural changes protruding from the visceral pleura were observed intraoperatively. This is of significant value because no other reports have described the appearance of the tumor during surgery.

On CT imaging, the nodules of PHG are often relatively well-defined, as demonstrated in our patients. However, some lesions present with spiculated nodules or cystic changes on CT [4], resembling lung cancer [5]. Mild FDG uptake is typically observed on PET-CT, although cases with an SUVmax in the range of 6 have been reported, thereby limiting its utility for diagnosis [3,6]. In Case 2, mild FDG uptake (SUVmax=3.0) was observed on PET-CT. Notably, no cases of PHG have reached a definitive diagnosis on preoperative biopsy or intraoperative frozen section alone.

Furthermore, it can be challenging to differentiate PHG from conditions such as lung cancer, metastatic tumors, or amyloidosis. For instance, a previous case misdiagnosed as PHG via CT-guided biopsy was later revealed as pulmonary involvement of Castleman disease through video-assisted thoracoscopic biopsy [7]. In this study, intraoperative frozen section indicated fibrosis in both patients, but the definitive diagnosis was only achieved after pathological evaluation of the wedge resection specimens. Thus, pathological evaluation of surgical specimens from thoracoscopic lung biopsy is necessary to arrive at a definitive diagnosis. When frozen section shows only fibrosis, a definitive intraoperative diagnosis of PHG is often difficult; to minimize misdiagnosis, clinicians should obtain multiple biopsy specimens and defer a final diagnosis to permanent pathological evaluation when uncertainty remains.

There is no established treatment for PHG. Although there are some case reports regarding the use of corticosteroids, their efficacy remains inconsistent, with both favorable and unfavorable outcomes documented [1,8-12]. Some studies have reported that over 15% of patients experienced nodule enlargement even during or after corticosteroid therapy. In contrast, other studies have shown that corticosteroid-treated patients had a significantly higher rate of nodule reduction on radiographic imaging versus untreated patients (42.1% versus 4.4%) [1]. In cases with a solitary nodule, surgical resection may be curative. Nevertheless, more than 60% of cases overall reportedly remain stable without any treatment [1]. In our patients, no nodule enlargement or emergence of new nodules was observed at around one year postoperatively.

The prognosis of PHG is generally favorable, although some reports have indicated a recurrence rate of 46% among patients who underwent curative resection [1]. One case report described a patient who was being considered for lung transplantation due to nodule enlargement leading to severe end-stage lung disease with severe obstructive and restrictive pulmonary dysfunction [12]. Another report described a patient who initiated home oxygen therapy for respiratory failure, but it was no longer required after successful lung transplantation [11]. These reports suggest the importance of careful follow-up for the development of new nodules and the coexistence of pulmonary malignancies. In our patients, no nodule enlargement or the appearance of new nodules has been observed to date, and regular follow-up is planned for these patients.

## Conclusions

The diagnosis of PHG remains challenging both preoperatively and intraoperatively. In the present case, the intraoperative frozen section was misdiagnosed as fibrosis, highlighting the difficulty of intraoperative evaluation. Thoracoscopic lung biopsy plays a crucial role in obtaining a definitive diagnosis. The difficulty of diagnosis, both preoperatively and intraoperatively, should be recognized not only by thoracic surgeons but also by all physicians involved in pulmonary care.
